# Grass pollen allergy in children and adolescents-symptoms, health related quality of life and the value of pollen prognosis

**DOI:** 10.1186/2045-7022-3-19

**Published:** 2013-06-22

**Authors:** Hampus Kiotseridis, Corrado M Cilio, Leif Bjermer, Alf Tunsäter, Helene Jacobsson, Åslög Dahl

**Affiliations:** 1 Pediatric Clinic, Malmö, Skåne University Hospital, Lund University, Lund, Sweden; 2 Departments of Respiratory Medicine and Allergology, Skåne University Hospital, Lund University, Lund, Sweden; 3 Competence Centre for Clinical Research, Skåne University Hospital, Lund, Sweden; 4 Departments of Biological and Environmental Sciences, Gothenburg University, Gothenburg, Sweden

**Keywords:** Grass pollen allergy, Rhinoconjunctivitis, Asthma, Quality of life, Children, Pollen forecasts

## Abstract

**Introduction:**

An association between pollen count (Poaceae) and symptoms is well known, but to a lesser degree the importance of priming and lag effects. Also, threshold levels for changes in symptom severity need to be validated. The present study aims to investigate the relationship between pollen counts, symptoms and health related quality of life (HRQL), and to validate thresholds levels, useful in public pollen warnings.

**Material and methods:**

Children aged 7–18 with grass pollen allergy filled out a symptom diary during the pollen season for nose, eyes and lung symptoms, as well as a HRQL questionnaire every week. Pollen counts were monitored using a volumetric spore trap.

**Results:**

89 (91%) of the included 98 children completed the study. There was a clear association between pollen count, symptom severity and HRQL during the whole pollen season, but no difference in this respect between early and late pollen season. There was a lag effect of 1–3 days after pollen exposure except for lung symptoms. We found only two threshold levels, at 30 and 80 pollen grains/m^3^ for the total symptom score, not three as is used today. The nose and eyes reacted to low doses, but for the lung symptoms, symptom strength did hardly change until 50 pollen grains/m^3^.

**Conclusion:**

Grass pollen has an effect on symptoms and HRQL, lasting up to 5 days after exposure. Symptoms from the lungs appear to have higher threshold levels than the eyes and the nose. Overall symptom severity does not appear to change during the course of season. Threshold levels need to be revised. We suggest a traffic light model for public pollen warnings directed to children, where green signifies “no problem”, yellow signifies “can be problems, especially if you are highly sensitive” and red signifies “alert – take action”.

## Introduction

Allergic diseases like rhinoconjunctivitis, asthma and eczema are major health problems in the western population. Although the diseases traditionally defined by the major target organ of the allergic inflammation, they are tightly linked. The allergic inflammation has components that can give rise to systemic disease manifestations [1], [2] E.g., 80 per cent of children with atopic dermatitis will eventually have asthma or rhinitis [3]. 30 per cent of children with rhinitis have asthma, and more than 80 per cent of children with asthma have rhinitis symptoms [4].

Airborne pollen is one of the most common triggers of allergic disease. They are formed by flowering plants, among which some are especially prone cause to allergic disease [5]. These pollen grains contain allergens that trigger the allergic inflammatory response via mast cell activation in the human mucosa of the target organs, which then give rise to a systemic reaction.

Pollen that gives rise to human disease is present in the air in northern Europe at least 6 months every year. This fact has great implications for children affected, and interferes in many aspects of their daily lives - not only physical and practical but also emotional [6]. The HRQL is an important aspect that should be taken into account in the clinical follow up and is recommended to be included in clinical studies [7]. Atmospheric pollen counts are considered to be positively correlated with allergic symptomatology [8]. This has then been confirmed in numerous studies [9-12] but the delay of symptoms after the exposure, the impact of earlier exposure (priming and lag effects) and possible changes in reaction patterns during the course of the season have been less often described, just as differences in reaction patterns between different organs.

The first line treatment in respiratory allergy is allergy avoidance [13]. Pollen warnings are meant to be part of a guided self-management to prevent system aggravation and help the allergy sufferers take the control of the condition. They increase awareness about the disease and its connection to ambient aeroallergen levels, and thus act in patient education. Pollen warnings should be presented to the public in a structured, easily understandable way.

The aim was to study the effect of grass pollen exposure on symptoms and HRQL during the grass pollen season, and to validate the threshold values for pollen concentration, used in pollen warnings for children.

## Material and methods

Children aged 7–18 with grass pollen allergy were consecutively selected from May 2009-01-01 until 2009-03-31. The majority of patients included in the study lived in the inner city of Malmö. Some lived in the suburban areas. Most of the children were seeing a paediatrician because of their allergy. Most of them were seeking help because of rhinoconjunctivitis, asthma or both.

The diagnosis of grass pollen allergy was, in addition to the clinical history, ascertained by a positive skin prick test or by the presence of allergy specific IgE in the blood.

Patients were excluded if they had allergic symptoms caused by other allergens during the grass pollen season.

The children filled out a HRQL questionnaire at the end of the week for every week until mid-July. Every day during the study period (1^st^ June – 12^rd^ July) they filled out a diary for symptom severity. All were completed by the children themselves. The study was approved by the local ethics committee and informed consent was obtained from parents and subjects.

The study protocol was approved by the Coordinating Ethics Committee of Lund University.

### Severity assessment

For the classification of rhinitis we used the ARIA (Allergic Rhinitis and its Impact on Asthma) guidelines [13]. These guidelines classify rhinitis as intermittent allergic rhinitis or persistent allergic rhinitis (PAR), on the basis of the duration of symptoms. The ARIA classification also classifies the severity on the basis of the presence or absence of impairment in any of 4 health-related quality of life (HRQL) items: *sleep*, *daily activities*/*sport*, *work*/*school*, and *troublesome symptoms*. According to guidelines the rhinitis was defined as mild when there was no impairment in any of these items, and moderate/severe when there was impairment in 1 or more areas [13].

For the asthma severity assessment the guidelines of the Swedish society of paediatric allergology based on medication level were used [14].

### HRQL

As a measure of HRQL the Pediatric allergic disease quality of life questionnaire (PADQLQ) was used. The total score covers three domains: a practical a physical and an emotional domain. The questions are answered with a figure (0–6) where 0 means “not troubled at all” and 6 means extremely troubled. The minimal important difference of the total score (0–6) is 0.2[15]. The instrument is validated and used in English and Swedish [16], [17] The questions were answered by the child.

### Symptom scores

The children used a symptom diary for assessing severity on a daily basis on symptoms from eye, nose and lungs. The severity scale included four categories: no symptoms (0), mild symptoms [1], moderate symptoms [2] and severe symptoms [3].

### Pollen counts

Daily atmospheric pollen counts for the Malmö area, comprising mainly urban and agricultural land in the North-European nemoral vegetation zone, were monitored during the pollen season 2009 using a Burkard 7-day volumetric spore trap, situated at a roof top about 25 m above ground at Skåne University Hospital (SUS), 55˚60’N, 13˚00’E. The exposed tapes were analysed by Botaniska Analysgruppen i Göteborg AB. The counts are representative for an area with a radius of 30 km from the trap, encompassing the residence of all subjects in the study.

### Statistical methods

To estimate the association between pollen counts symptoms, HRQL and lag effects mixed models on repeated observations were performed. Three different symptoms (nose, eye and lung), the mean of the three different symptoms (total) and HRQL were used as outcome. The outcomes were on ordinal scales; the symptoms 0–3 and HRQL 0–6. The pollen counts were tested as fixed effect and were analysed both as continuous and categorical variables (threshold levels). Lag effects were also tested. The analyses were performed on the whole period (day 1–42) and on three sub-periods (day 8–17, 18–27 and day 28–37). The change in reactivity to pollen exposure during the pollen season were analyzed for two periods (day 1–7 and 36–42). Period (1 = day 1–7 and 2 = 36-42) was added to the model. The estimates were obtained by the procedure GENMOD in SAS.

Breaking points for the symptom score (nose, eye, lung and total) were obtained by the fitting method Loess in SPSS. 50% of points to fit and kernel Epanechnikov were used.

The statistical analyses were performed in SPSS Statistics 18 for Windows (IBM Corporation, Somers, NY, USA) and SAS 9.2 for Windows (SAS Institute Inc., Cary, NC, USA). A p-value below 0.05 was considered statistically significant.

## Results

### Patient population

98 children aged 7–18 years were enrolled into the study. 89 (91%) completed the study. 89.8% had seasonal allergic rhinoconjunctivitis and 60.2% suffered from seasonal allergic asthma. Of the patients with rhinitis, 85.1% was classified as moderate/severe. A majority of the rhinitis patients also had concomitant asthma (61.5%) (Table 1).

**Table 1:** Subject characteristics

**Diagnosis**	**Median (min–max) or**	**Missing values**
**Characteristic**	**%**	**n**
Rhinoconjunctivitis and asthma (n = 89)		
Age	13 (7–18)	0
Rhinitis	89.8	1
Food allergy	39.1	2
Asthma	60.2	1
Eczema	37.2	3
Rhinoconjunctivitis (n = 79)		
Classification		5
Mild intermittent	1.4	
Mild persistent	0.0	
Moderate/severe intermittent	13.5	
Moderate/severe persistent	85.1	
Asthma	61.5	1
Affected sleep	71.2	6
Affected school performance	62.9	9
Troublesome symptoms	97.3	5
Daily activity	81.7	8
Multiple sensitisation	61.1	7
Immunotherapy	3.8	0
Oral steroid	0.0	0
Antihistamine	93.7	0
Antihistamine nose	16.5	0
Nasal steroid	53.2	0
Antihistamine eye	45.6	0
Cromoglycate eye	28.2	1
Asthma (n = 53)		
Asthma grade		0
1	18.9	
2	24.5	
3	56.6	
Rhinitis	92.3	1
ICS	90.6	0
Antileukotriene	18.9	0

### The pollen season

**Figure 1:**
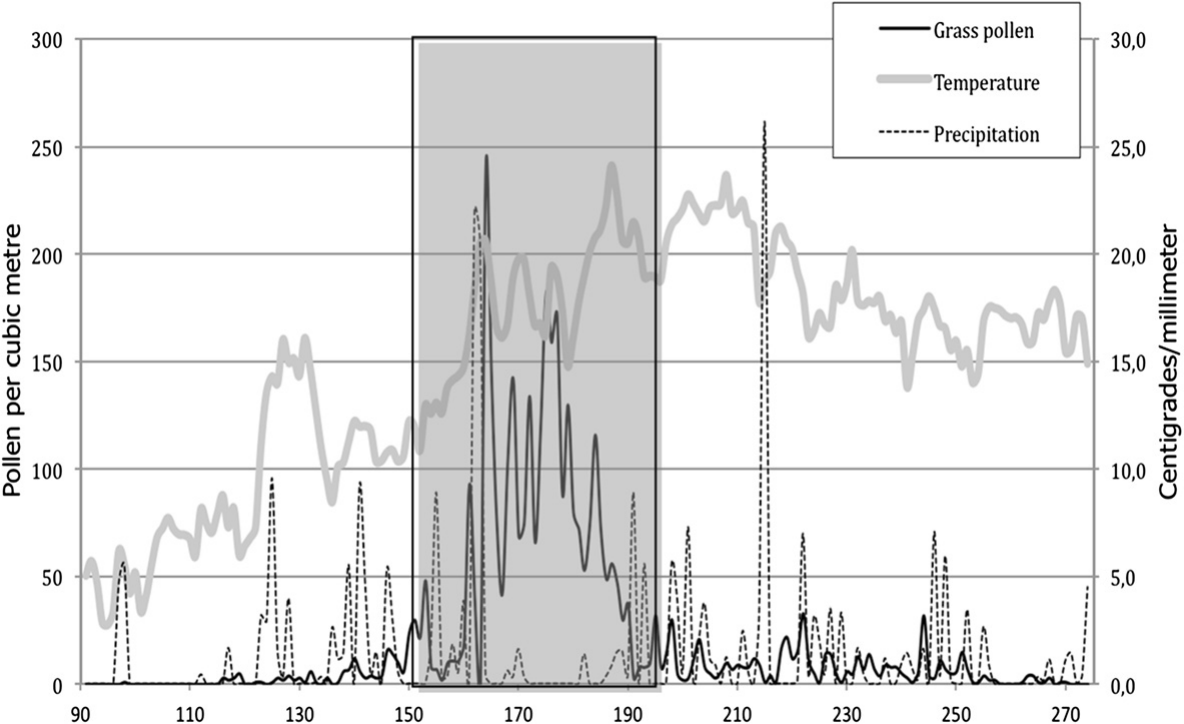
**Daily pollen grains**, **maximum temperatures and precipitation in Malmö 2009.** The study period 1 June–12 July (ordinal dates 152–193) is delimited with a pale blue square.

The first grass pollen was registered 26 April (Figure 1). From 5 May, grass pollen was registered on five days on a row. On 25 May, the grass pollen count was 4 pollen grains per cubic meter, but then, counts started to increase and on 31 May, the day before the start of the study period the pollen count was 30 pollen grains/m^3^. During the study period, the pollen count varied between 1 and 242 pollen/m^3^. The total number of registered pollen during the study period was 2920, i.e. 79% of the total pollen index (3700) during the entire grass flowering period. The study period encompassed most of the anthesis of species belonging to the subfamily Pooideae, which are the main provokers of grass pollen induced allergy in Sweden [18]. The peak occurred at 13 June (Figure 1), the day after a heavy rain, and counts then did not decrease below 50 grains/m^3^ until 5 July. The values for the 1^st^ quartile, median, and 3^rd^ quartile were 17, 61, and 107 pollen grains per cubic meter, respectively. After the end of the study period, the maximum daily count was 33 pollen/m^3^, and the weather turned cooler with more precipitation.

### Relationship between pollen count and symptoms

**Figure 2:**
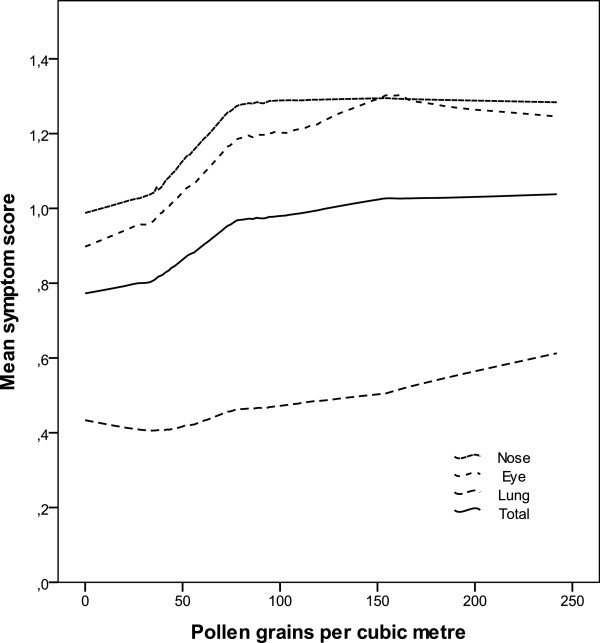
**The relationship between symptom scores and pollen grains during the study period 1 June**–**12 July in Malmö 2009**, **evaluated with locally weighted regression (LOESS).**

The breaking points for total symptom aggravation were visually inferred from a Loess curve (Figure 2). We found two sharp inflexion points at 30 and 80 pollen/m^3^, and a less clear one at 150 pollen/m^3^. There was a significant relationship for the total symptom scores on pollen count when calculated for the entire study period (p < 0.0001), the period comprising study days 8–17 (p = 0.0011), and for the period comprising study days 28–37 (p = 0.037).

*Nose symptom scores* increased continuously and linearly with pollen counts from concentrations of 0–30 grains/m^3^ (Figure 2), wherefrom symptom severity increased faster until about 80 pollen/m^3^. At higher pollen concentrations, the severity of symptoms did not appear to increase. There was a dependence on pollen count during the entire study period, during days 1–42 and during days 8–17 (for both, p < 0.0001). During the period days 28–37, the dependence was near significance (p = 0.066).

*Eye symptom scores* increased with pollen count beyond concentrations of about 70 grains/m^3^ and did not level out until about 140–150 pollen/m^3^ Two lower inflexion points of the curve were located at pollen concentrations of about 30 and about 80 pollen/m^3^ (Figure 2). The dependence of symptoms on pollen count was significant when calculated for the entire study period (p < 0.0001). For the final period (days 28–37), the relationship was near significance (p = 0.074).

*Lung symptoms scores* related to increasing pollen counts in a pattern different from that of the nose and the eyes (Figure 2). There was no apparent change in symptom severity until levels above 50 pollen/m^3^. From levels above about 70 pollen/m^3^, the increase appeared almost linear. During the period days 1–42, there was a dependence of symptoms on pollen counts (p = 0.0025).

### Lag effects, accumulation of exposure effects and symptom severity

Pollen exposure had a significant effect on nose, eyes and lung symptoms. With an increasing lag of 1–3 days, significance levels decreased. For lung symptoms, the effect was nearly significant (p = 0.055) for a lag of one day but with two or three days, there was none. For nose and eye symptoms, the effect of exposure 3 days before symptom registration was still significant, but significance level had decreased from p < 0.0001 for a lag of 0–2 days to p = 0.0021 for nose and p = 0.0007 for eyes. For total symptoms, the significance level at a lag of 3 days decreased to p = 0.0014.

If the day when symptoms were registered was excluded, and only accumulated pollen sum during three days preceding this date was included, there was still a strongly significant effect on nose, eye and total symptoms (p < 0.0001), but none on lung symptoms. With an extension to five days before registration day, the effect remained strongly significant for eye symptoms, and significant to a lower degree for nose and total symptoms (p = 0.0004, and p = 0.0001, respectively).

### Reactivity late in pollen season

The change in reactivity to pollen exposure during the pollen season was analysed for two periods (day 1–7 and day 36–42). The change in symptom severity by pollen exposure did not change significantly during the season neither for nose, eyes, lungs and total symptom score.

### Threshold levels

**Figure 3:**
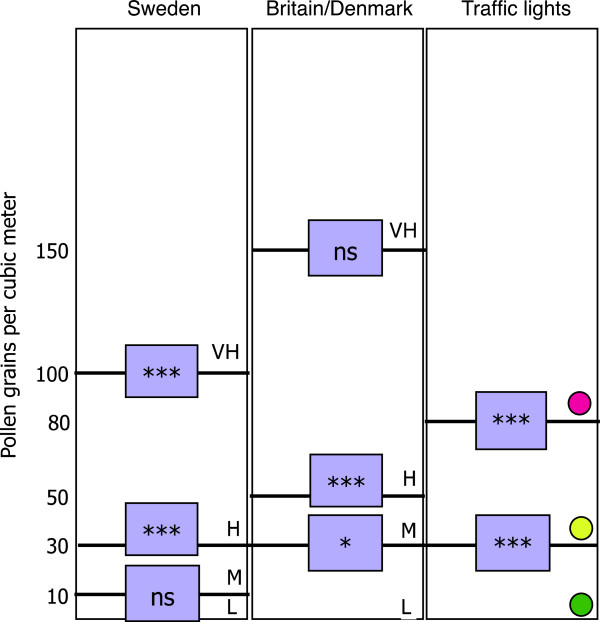
**Threshold levels in Sweden and Britain/Denmark (L = Low, M = Medium, H = High and VH = Very High), and the traffic light model (Low, Medium and High).** There was no significant change (ns) in symptom score between low and medium levels, when the Swedish threshold levels were used, neither was the change in symptom scores between high and very high levels as defined in the British system. * = p < 0.05, ** = p < 0.01, *** = p < 0.001.

We considered the nose, eye, lung and total symptom scores separately, and related symptom scores to Swedish and British/Danish pollen warning threshold levels (Table 2). We related them to a three-level alert system defined from the visual inference of the Loess curve (Figure 2), which we call the “traffic light system” with green, yellow, and red light, in order to easily communicate the symptom risk level to children (Figure 3).

**Table 2:** Threshold levels

**Symptom**	**Threshold level**	**Pollen grain per cubic metre level**	**OR (95% CI)**	**P-value**
Nose	Sweden	Low–Medium	0.98(0.84–1.13)	0.74
		Medium–High	1.42(1.19–1.69)	<0.0001
		High–Very high	1.24(1.07–1.44)	0.0049
	Britain/Denmark	Low–Medium	1.18(1.02–1.37)	0.026
		Medium–High	1.37(1.16–1.61)	0.0002
		High–Very high	1.04(0.88–1.24)	0.64
	Traffic lights	Low–Medium	1.40(1.19–1.65)	<0.0001
		Medium–High	1.19(1.02–1.40)	0.027
Eye	Sweden	Low–Medium	1.02(0.89–1.18)	0.77
		Medium–High	1.34(1.10–1.61)	0.028
		High–Very high	1.30(1.14–1.48)	<0.0001
	Britain/Denmark	Low–Medium	1.12(0.96–1.31)	0.15
		Medium–High	1.38(1.17–1.62)	<0.0001
		High–Very high	1.30(1.09–1.56)	0.0038
	Traffic lights	Low–Medium	1.33(1.13–1.56)	0.0007
		Medium–High	1.30(1.14–1.48)	<0.0001
Lung	Sweden	Low–Medium	0.92(0.76–1.10)	0.35
		Medium–High	1.10(0.89–1.35)	0.39
		High–Very high	1.27(1.13–1.43)	<0.0001
	Britain/Denmark	Low–Medium	0.95(0.83–1.10)	0.51
		Medium–High	1.21(1.03–1.43)	0.020
		High–Very high	1.20(1.01–1.42)	0.041
	Traffic lights	Low–Medium	1.02(0.85–1.23)	0.82
		Medium–High	1.27(1.12–1.45)	0.0003
Total	Sweden	Low–Medium	1.04(0.91–1.18)	0.57
		Medium–High	1.35(1.15–1.59)	0.0003
		High–Very high	1.25(1.11–1.42)	0.0004
	Britain/Denmark	Low–Medium	1.14(1.00–1.29)	0.046
		Medium–High	1.39(1.20–1.62)	<0.0001
		High–Very high	1.14(0.96–1.35)	0.12
	Traffic lights	Low–Medium	1.36(1.18–1.58)	<0.0001
		Medium–High	1.25(1.10–1.42)	0.0008

### Lag effects, accumulation of exposure effects, and estimation of HRQL

**Figure 4:**
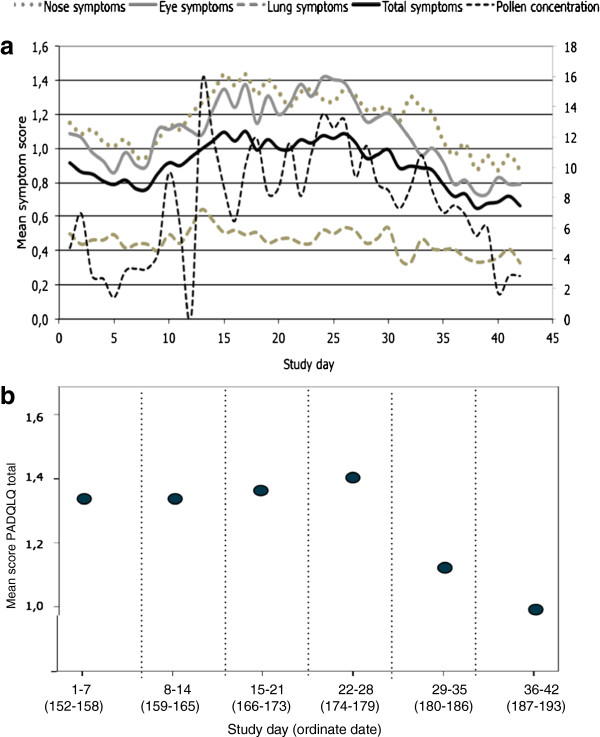
**Symptoms and HRQL during grass pollen season. a**. Mean symptom scores and square roots of grass pollen grains during the study period 1^st^ June–12^th^ July in Malmö 2009. **b**. Mean total PADQLQ score during the study period. The vertical lines delimit the weeks.

The mean PADQLQ score during the first study week was 1,35 and then increased to 1.4 during the fourth week Thereafter, the estimated scores decreased, to 1.1 during the fifth week (29^th^ June-5^th^ July, days 28–35) and 1.0 during the last week (6^th^ July-12^th^ July, days 36–42), concomitant with a generally decreasing trend in pollen concentration (Figure 4).

Pollen exposure during the days previous to the day when HRQL was estimated had an effect on the estimate, but less so, the more days that were included, and if the pollen counts on the estimation day was excluded. The strongest effect was found for the effect of the pollen count with a lag of 1 day (p = 0.0022). Pollen count during the single days 2 days, and 3 days before estimation day respectively, had no significant effects. The accumulated effect of four days, including registration day, or of 6 days, including estimation day, was significant (p = 0.0024, and p = 0.0034, respectively). If estimation day was excluded, and 3 days accumulated exposure or 5 days accumulated exposure were considered, the effects had significance levels of p = 0.0087 and p = 0.01, respectively. Mean pollen count during an entire week, estimation day included (days 0–6) had a significant effect (p = 0.010) on the estimation of HRQL score.

### Symptom severity over time

**Figure 5:**
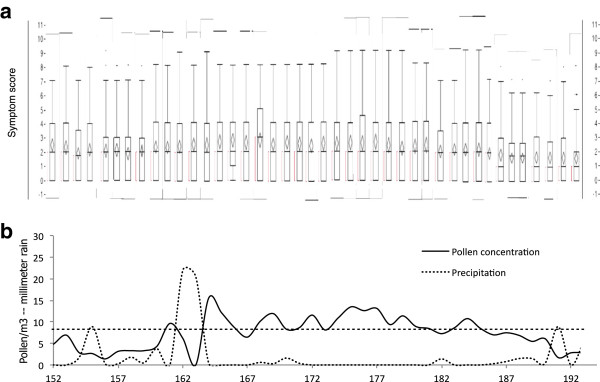
**a. Distribution of total symptom scores for each study day during the period 1 June–12 July in Malmö 2009.** Each box plot is composed of 3 horizontal lines displaying the 25th, 50th, 75 th and 90th percentiles. The dots represent outliers. **b**. Sum of pollen counts and precipitation, day by day during the same period. The horizontal line denotes 80 pollen grains per cubic metre, the suggested lower limit for “high grass pollen concentration”. Pollen counts are expressed as square roots of the actual numbers.

During the first ten days of the study, maximum symptom scores varied between 7 and 8. They varied between 8 and 9 during the period 8–28 June (when pollen concentration levels generally was higher than 65 pollen/m^3^, with exception for one day with heavy rain and another one, with 42 pollen/m^3^. Score sums of 9 were recorded also towards the end of this period, and also a couple of days later, when pollen concentration levels temporarily rose again. Thereafter, maximum scores varied between 6 and 7, and the very last day, reached 5 (Figure 5).

## Discussion

Pollen elicits symptoms in about 1 of every five persons, with a great effect both on individuals and on the society. Many people suffer in their daily lives, not being able to live the life they want, with a great effect on the quality of life. Furthermore, in the society, there are great costs for health care and medicine, and also a lot of indirect costs with absence from work and school.

Pollen affects human beings with allergic sensitisation since they carry allergens. In sensitized children, these allergens elicit an allergic reaction in the target organ and give rise to a systemic inflammation [19]. It has recently been demonstrated that pollen grains, under physiological exposure conditions, release not only allergens but also bioactive lipids and enzymes that activate human neutrophils and eosinophils in vitro [20], [21]. In other studies, atmospheric pollen count were found to be positively correlated with allergic symptoms, drug consumption for allergic rhinitis and/or conjunctivitis [9-12], emergency visits because of asthma [22-26], and hospitalizations because of asthma [27-29]. However, not only sensitization rates but also the severity of reactions to the same pollen concentrations may vary between different regions [30]. Thus, it is important to investigate these patterns in different populations. Moreover, most studies have so far not investigated if the reactions change with time, and either focus on one target organ or lump symptoms from several organs together. In the present study, we have found a strong relation between symptoms, quality of life and grass pollen exposure. We also found that different organs react differently when pollen concentrations change, also with regard to the effect of exposure one to three days before assessment day. There is a lag effect of the preceding days upon eye and nose, but not upon lung symptoms. We did not find any signs of aggravation, nor of symptom relief over time that could not be related to increasing or decreasing pollen levels on the assessment day or on the preceding three-day period.

Citizens who are well informed on factors in their environment, e.g., the presence of aerosols with a possible adverse effect on health, are able to take measures in order to protect themselves from these negative effects. According to EU directives, this information should not only comprise anthropogenic, but also natural sources of such aerosols [31], [32]. Information about registered and forecasted amounts of allergenic and airborne pollen helps the allergy sufferer to identify his or her disease and relate it to ambient concentration levels. The allergic person can avoid activities that enhance exposure risk or demand a high level of concentration and precision, and may take appropriate medication to reduce the effects of exposure. Thus, he or she may take control of the disease, with increased performance and quality of life.

In many countries and regions, this information service is already running. Pollen concentration is measured at a daily basis, and the registered numbers and/or short-term forecasts are usually presented in media and special web sites, usually translated to categories such as low, moderate and high levels. The delimitation of these categories varies from country to country. They may either be determined according to the number of individuals that experience symptoms at a certain ambient pollen load [33], according to the severity of symptoms in an “average” allergic person, or to the general abundance of the pollen types [34]. In Malmö and other parts of Sweden, the categories used in the public warning system (Table 3) were delimitated in the 1970’s, referring to “clinical experience”. The capital of Denmark, Copenhagen is situated only a few kilometres from Malmö, across the strait of Öresund, and a considerable amount of inhabitants commute back and forth every day. However, in Copenhagen, the British threshold levels (Table 2) are used in public communication [35]. From an educational point of view, this situation is not desirable; first, the threshold levels should be clinically relevant, and second, the information given should not be confusing. Therefore, we wanted to investigate which of these systems, if any, reflects the clinical reactivity best.

**Table 3:** Odds Ratios for symptoms at different threshold levels between different pollen grain levels

	**Threshold level (pollen grains per cubic metre)**		
**Levels**	**Britain**/**Denmark**	**Sweden**	**Traffic lights**
Low	1–30	0–10	0–30
Moderate	31–50	11–30	31–80
High	51–150	31–100	81–
Very High	150–	101–	

In the children of our study group, we found three relevant levels reflecting the reaction towards grass pollen: at 0–30 pollen per cubic meter, giving no or minor symptoms, at 30–80 pollen per cubic meter giving intermediate symptoms, and then at more than 80 pollen per cubic meter causing severe symptoms (Figure 2). We found that the Swedish system has a unnecessary limit between “low” and “moderate” values at 10 pollen/m3, and that the British/Danish system includes a limit between “high” and “very high” levels that do not reflect any significant differences in symptom severity (Figure 3). We found that symptoms reached a plateau at 80 pollen/m3, and we do not believe that it is clinical meaningful to further categorize into very high levels. A similar plateau beyond 80–90 pollen grains was found from France and Switzerland, which suggests that the pollen limits found in our study might be applicable in northern and central Europe [36].

Most studies on the effects of bio aerosols on health focus on asthma exacerbations, measured as emergency visits or hospitalizations, or on rhinoconjunctivitis. Fewer consider symptoms from several organs at one time. We studied total symptom scores and nose, eye and lung symptoms separately and found that the two former and the latter vary with pollen concentration in different ways. The curves describing nose and eye symptoms are steeper and have a number of more or less sharp inflexion points, whereas the lung symptoms do not increase until pollen concentration reaches about 70 pollen per cubic meter, and the symptom scores where fairly low throughout our study (Figure 2). It is possible that pollen counts must be higher to have a clear effect on lung symptoms, or that exacerbations of such symptoms are associated with special meteorological conditions, such as humid conditions and thunderstorms that may cause the pollen grains to burst. Epidemiological studies have shown an association between even lower levels of pollen concentrations (less than 30 pollen grains) and hospital admissions, which suggests that these levels might have effect on susceptible individuals [37]. During the present study, there was little precipitation, with exception of one day just before the pollen peak, and the connection between heavy rains and lung symptoms could not be evaluated. But pollen-derived debris and small particles can be associated with pollen allergens, and since they are much smaller than the intact grains are themselves, they are able to penetrate into the lower airways as to induce asthma [38]. Furthermore, asthma is a complex disease with many different phenotypes where pollen allergy is only one of many factors influencing asthma control [39]. From our results, it is apparent that even if less dramatic, rhinoconjunctivitis must not be neglected, since there is a clear effect on the well being and HRQL of the involved children (Figure 4).

We found a lag effect up to 5 days. Some previous studies have found a lag phase of 1–5 days [40], [41]. We could not find any difference in reactivity between the beginning and the end of the pollen season. As early as in the 1960’s, repeated pollen challenges were shown to increase nasal sensitivity to other allergens in experimental models. This was called Connell’s priming effect [42]. De Weger et al. [43] found that that allergic rhinitis symptoms at similar grass pollen concentrations were more severe in the early flowering season as compared to those in the late flowering season. They suggested that there is a natural potential to down-regulate the allergic response after repeated allergen exposure, similar to the effects of successful immunotherapy. In contrast to the results of that study, we could neither find evidence for symptom relief, nor aggravation, during the course of the study period that could not be related to changes in pollen levels. The strength of our design is that we do not have symptom aggravation in early grass pollen season due to actual other allergen exposure (e.g. birch pollen), since birch senstized children were excluded.

### Strengths and limitations of this study

The strength of our study is the well-characterized patient population, the wide range of symptom severity in the children included in the study, the careful follow up including HRQL and symptom diary with repeated measurements.

It would have been appropriate to have monosensitized children in order to study the effect of grass pollen exposure but this was not possible since most of our population are multisensitized. We believe that the results in the present study population with partially multisensitized but only symptomatic to grass pollen are valid although we cannot exclude an underlying allergic inflammation affecting the result. Also a control group would have been preferred. In this study the children are their own controls. This has been done by assessment both in and out of season. The generalizability is limited to some degree since the recruitment of patients was made on patients seeking help for their pollen allergy and thus probably not representative for the whole grass pollen allergy population. We believe that the study population are representative for the population seeking help for their grass pollen allergy and thus is of great importance to study for the optimization of their treatment.

Other factors than pollen, such as humidity, temperature and air pollution, might also influence the result. Air pollution may be an important factor working synergistically with pollen eliciting symptoms. Air pollutants can both interact with pollen grains, leading to an increased release of antigens characterized by modified allergenicity, and affect the airways, by enhancing the contact between allergens and immunoactive cells, and thus reinforce allergic inflammation [31]. Particulate pollutants can interact with allergen-carrying paucimicronic particles derived from plants. The paucimicronic particles, pollen-originated or not, are able to reach peripheral airways with inhaled air, so inducing asthma in sensitized subjects [38].

Grass pollen originates from several species, which are not readily distinguished in the traditional pollen analysis. Most grass pollen allergies in Scandinavia are said to be induced by several species within the subfamily Pooideae, with extensive cross-allergy, but species belonging to other subfamilies are also present, e.g. *Phragmites*, although the peak of their flowering is later. When considering the effects of grass pollen, it would be optimal to have access to more detailed phenological information and measurements of airborne allergenic proteins, to be able explain if changes in symptoms is related to allergen load or not. However, our study is likely to have encompassed most of the Pooideae flowering period and the results should reflect such a relationship. Reactivity to other pollens, like birch, still has to be validated.

Another limitation is the lack of medication score. The effect of pollen exposure should also include the medication need, which can affect both symptoms and HRQL. This is recommended in guidelines for clinical studies[7]. In the present observational study, children had a wide range of different medications and most of them used their medication regularly and thus are not believed to correlate to the burden of pollen exposure.

All the analyses presented here are conducted on group level. Web-based individual based forecasts exists, where the patient fills in a diary to estimate the sensitivity to pollen exposure will be useful, but need to be developed for optimal communication with different target groups and to suit their varying reaction patterns according to age and geographical origin.

## Conclusion

Respiratory allergy is a great problem, and in case of grass pollen allergy we find an effect on symptoms and on HRQL, lasting up to 5 days after exposure. We neither find indications of symptom aggravation, nor alleviation during the course of exposure. One cornerstone in treatment in respiratory allergy and in pollen allergy is the availability of pollen forecasts to make it possible to take preventive actions. Such a system should be evidence based, and easy to understand. We suggest a traffic light model for public pollen warnings directed to children, where green means “no problem”, yellow means “can be problems, especially if you are highly sensitive” and red means “alert – take action”.

## Abbreviations

PADQLQ: Pediatric allergic disease quality of life questionnaire; QoL: Quality of life; HRQL: Health related quality of life.

## Competing interests

The authors have stated explicitly that there are no conflicts of interest.

## Authors contributions

HK initiated, designed and performed the study, collected and analysed the data and wrote the paper. CC, LB, and contributed to the design of study and writing of the paper. HJ co-analysed the data and wrote the paper. AT and ÅD contributed to the design of the study and analysis of data and the writing of the paper. All authors read and approved the final manuscript.
